# Green synthesis as a simple and rapid route to protein modified magnetic nanoparticles for use in the development of a fluorometric molecularly imprinted polymer-based assay for detection of myoglobin

**DOI:** 10.1088/1361-6528/abce2d

**Published:** 2020-12-10

**Authors:** Mark V Sullivan, William J Stockburn, Philippa C Hawes, Tim Mercer, Subrayal M Reddy

**Affiliations:** 1 Research Centre for Smart Materials, Department of Chemistry, School of Natural Sciences, University of Central Lancashire, Preston, PR1 2HE, United Kingdom; 2 Leicester School of Pharmacy, De Montford University, The Gateway, Leicester, LE1 9BH, United Kingdom; 3 Division of Forensic and Applied Sciences, School of Natural Sciences, University of Central Lancashire, Preston, PR1 2HE, United Kingdom; 4 The Pirbright Institute, Ash Road, Pirbright, Woking, Surrey, GU24 0NF, United Kingdom; 5 Jeremiah Horrocks Institute for Mathematics, Physics and Astronomy, School of Natural Sciences University of Central Lancashire, Preston, PR1 2HE, United Kingdom

**Keywords:** molecularly imprinted polymers, fluorescent MIP, fluorescent assay, microwave, green chemistry, magnetic nanomaterials

## Abstract

We have developed a low-cost molecularly imprinted polymer (MIP)-based fluorometric assay to directly quantify myoglobin in a biological sample. The assay uses a previously unreported method for the development of microwave-assisted rapid synthesis of aldehyde functionalized magnetic nanoparticles, in just 20 min. The aldehyde functionalized nanoparticles have an average size of 7.5 nm ± 1.8 and saturation magnetizations of 31.8 emu g^−1^ with near-closed magnetization loops, confirming their superparamagnetic properties. We have subsequently shown that protein tethering was possible to the aldehyde particles, with 0.25 ± 0.013 mg of myoglobin adsorbed to 20 mg of the nanomaterial. Myoglobin-specific fluorescently tagged MIP (F-MIP) particles were synthesized and used within the assay to capture myoglobin from a test sample. Excess F-MIP was removed from the sample using protein functionalized magnetic nanoparticles (Mb-SPION), with the remaining sample analyzed using fluorescence spectroscopy. The obtained calibration plot of myoglobin showed a linear correlation ranging from 60 pg ml^−1^ to 6 mg ml^−1^ with the limit of detection of 60 pg ml^−1^. This method was successfully used to detect myoglobin in spiked fetal calf serum, with a recovery rate of more than 93%.

## Introduction

A biomarker is a characteristic that is accurately measured and evaluated as an indicator of normal biological processes, pathogenic processes, or pharmacologic responses to a therapeutic intervention [1]. They are usually measured from tissue biopsies or liquid (blood, saliva, and urine). Biomarkers are critical to the development of drug delivery and medical devices [2] and are currently being used in research and clinical practice for:

• Diagnosing diseases or predicting risks of diseases.
• Monitoring healthy people to detect early signs of a disease.
• Determining whether a treatment is efficient or not.
• Targeting groups of people for whom a certain drug may be useful.
• Producing safer drugs by predicting the potential adverse effects earlier [3, 4].

Traditional biomarkers of myocardial necrosis are cardiac enzymes (kinase), myoglobin (Mb) and troponin [5–7]. Myoglobin is the earliest biomarker to appear, following acute myocardial infarction (AMI) [8]. It is an important oxygen carrying monomeric heme-protein that is found in heart and skeletal muscles, with its main function being the uptake of oxygen from oxyhemoglobin in the bloodstream [9]. When muscle cells are damaged Mb is rapidly released into blood circulation. Mb that is determined in serum can be used as an important biomarker for cardiac injury [10]. The normal range of Mb present in the serum of healthy patients is usually between 0 and 85 ng ml^−1^ [11]. The concentration of Mb within serum can quickly become elevated in as little as one hour after AMI and this correlates with the degree of myocardial injury [12, 13]. Current clinical practises use immunoassays, for the detection of AMI biomarkers in blood serum, which have relatively low accuracy and sensitivity, making the monitoring of disease treatment difficult. Work by Zaninotto, *et al* show that the BNA and OPUS assay have relatively high imprecision (>7.4%), as does the Access assay (6.0%–11.0%) [14]. There is, therefore scope for the development of new and more accurate assays and diagnostics.

Fluorescence assays, work by measuring emission intensity generated from a sample, the emission intensity being directly proportional to the concentration of the fluorophore present [15]. A typical method used for biomarker detection in solution is to use a heterogeneous non-competitive assay. This is when the analyte, within the sample, binds with labeled capture molecules. Leftover unbound labeled capture molecules are then removed and then the remaining bound labeled capture molecules are measured. The intensity of the signal is directly proportional to the concentration of a known analyte [16]. Fluorescence methods are one of the most widespread label-based approaches used in biomarker detection [17]. This is because of the availability of many fluorescent probes with diverse spectral properties, use of visible light, and rapid detection and signal generation. Affinity-based methods (immunoassays and hybridization assays) that rely on the ability of biomolecules (antibodies and nucleic acids) to specifically recognize and bind to target molecules, are used in conjunction with label-based biomarker detection [18]. While, being well-established and highly efficient, this diagnostic method routinely uses antibodies and enzymes, because of their sensitivity and affinity. Unfortunately, they still suffer from short shelf-life, high manufacturing cost and relatively poor stability (especially in organic solvents, and at extreme temperature and pH values) [19]. Additionally, it can often be difficult to immobilize antibodies on to the supports that are currently being used in diagnostic assays [20].

In order for a diagnostic test to be efficient, parameters such as specificity, cost, limits of detection (LOD) and analysis time are very important [21]. As an alternative to the antibody approach, molecularly imprinted polymers (MIPs) have been used in non-competitive assays and biosensors, essentially as capture molecules. Molecular imprinting is a technique used to synthesize template-shaped cavities within a polymer matrix with affinity to a chosen ‘template’ molecule. The process involves initiating the polymerization of monomers in the presence of a template molecule [22–24]. The template is then extracted, leaving a polymer with an affinity for the original template molecule [25, 26]. While MIPs may currently lack sensitivity for efficient application for early disease diagnosis they can be employed in the screening and monitoring stages of a disease [27]. MIP-based biosensors have been reported for protein biomarkers, including PSA, alpha-fetoprotein (AFP), and carcinoembryonic antigen (CEA), with lower detection limits of 9.6 × 10^–3^, 1.25 × 10^–3^, and 8 ng ml^−1^, respectively [28–30]. Erturk *et al* showed that while these LODs are good, they are yet to compete with similar, antibody-based assays suggesting there is possibility for improvement [28]. The use of the whole protein as a template will increase the accuracy of the MIP, although the same imprinted sites formed for these larger structures could potentially be attractive sites for similar sized or smaller protein binding (due to other proteins native to a blood sample), leading to unwanted cross-reactivity and reduced selectivity [31]. When compared with the current protein detection methods (electrochemistry, ELISA, SPR, and colorimetric and fluorescence assays), significant advances still need to be made with MIP-based sensors, with regards to the sensitivity, selectivity and the capacity for multi-analyte detection [21, 31]. Fluorescence materials can be incorporated into sensors and fluorescence tagged MIP-based sensors offer a convenient solution due to increased sensitivity and selectivity, while also offering many advantages such as short analysis time, ease of use and small sample volume [32]. Adding a fluorescent monomer during MIP synthesis is potentially an easy way to prepare a fluorescent MIP (F-MIP). For example, Ashley *et al* (2018) used fluorescein-*O-*acrylate as the fluorescent tag, with fluorescent quenching used in the detection of low molecular weight doxycycline [27, 33]. This suggests that using a fluorescent tag with a MIP imprinted for a biomarker could be a successful approach.

Superparamagnetic iron oxide nanoparticles (SPIONs) are particles of iron oxide with a diameter between 1 and 100 nm [34, 35] and are available in the two main forms of: magnetite (Fe_3_O_4_) and maghemite (*γ*-Fe_2_O_3_). They are created when ferrimagnetic, multidomain samples of iron oxide (Fe_3_O_4_ and *γ*-Fe_2_O_3_) particles are reduced to sizes less than around 100 nm [35]. SPIONs are able to show robust paramagnetic nature, with high sensitivity under the appliance of a magnetic field and complete disappearance of that nature once the magnetic field is removed; thus, removing any chance of magnetic remanence and coercivity. Due to their superparamagnetic properties and potential non-toxicity to humans, there has been an extensive amount of interest in the use and synthesis of SPIONs, especially within biological and medicinal applications. For biomedical applications these include magnetic resonance imaging, magnetic fluid hyperthermia, bioseparation (DNA, proteins and cells), biomolecule immobilization, and magnetic targeted drug delivery; amongst others [36–45]. SPIONs offer essential properties including magnetic susceptibility, low cytotoxicity, good biocompatibility, and stability under physiological environments [46].

In order for SPIONs to interact with bioactive substances, they are usually functionalized with a reactive group, typically amino (–NH_2_), aldehyde (–CHO) or carboxylic acid (–COOH) [47, 48]. These modified SPIONs can be used to bind to biomolecules, such as proteins, through non-covalent (hydrogen bonding) interactions but primarily through covalent linkages [47, 48]. They are preferred for use in biomedicine as they are biocompatible and potentially non-toxic to humans [46]. Iron oxide is easily degradable and can therefore be useful when used for *in vivo* applications [49] such as cancer magnetic therapy [50, 51]. Here the iron oxide nanoparticles are loaded with antitumor drugs and remotely directed via an external magnetic field. This allows for more accurate, localized targeting systems, as well as further enhancing the antitumor activity by moderate magnetic hyperthermia (below 40 °C) [40].

There have been several different methods used to produce SPIONs of a controlled size and shape with solvothermal methods proving particularly successful [52, 53]. However, solvothermal methods can be multi-step processes and time consuming (>8 h), producing only small yields of functionalized particles [48, 54, 55]. Recently, there has been a surge in interest in the application of microwave radiation as a thermodynamic driving force, with the prospect of developing environmentally conscious, simple and time efficient routes towards the synthesis of nanomaterials [56].

In this paper a heterogeneous non-competitive assay was developed for the detection of the AMI biomarker myoglobin. A F-MIP was created for the target molecule (myoglobin), with this MIP being used as a capture molecule within an assay for Mb. Previous work showed that acrylamide was an effective monomer to use within a MIP for myoglobin, because the monomer is water soluble, produces excellent rebinding, with minimal effect on the secondary structure of the protein [57]. The fluorescent monomer, fluorescein *O*-acrylate was chemically bound during myoglobin MIP synthesis to allow for fluorescence spectroscopy detection. The resulting F-MIP was added to a sample containing the target molecule, whereby the target molecule would bind to the MIP. Excess MIP would then be removed by the addition of myoglobin modified SPIONs, synthesized using a previously unreported microwave-assisted one-step 20 min synthesis. These particles would essentially bind to any excess MIP, which could then be separated using a magnet leaving only the target protein bound MIPs in suspension. The F-MIPs in suspension (bound to target protein) were then analyzed using fluorescence spectroscopy, whereby the intensity of the signal is proportional to the amount of target protein in the sample.

## Experimental

### Materials

7-[4-(Trifluoromethyl)coumarin]methacrylamide), acetonitrile, acrylamide (AAm), ammonium persulphate (APS), anhydrous sodium acetate (NaOAc), bovine serum albumin (BSA) ethanol, ethyl acetate, ethylene glycol, ferric chloride hexahydrate (FeCl_3_ · 6H_2_O), fetal calf serum (FCS), fluorescein *O*-acrylate, glacial acetic acid (AcOH), glutaraldehyde, myoglobin (Mb) (from equine skeletal muscle), *N N, N*′-methylenebisacrylamide (mBAm), phosphate buffered saline (PBS), sodium dodecyl sulfate (SDS), tetramethylethyldiamide (TEMED), were all purchased and used without purification from Sigma-Aldrich, Poole, Dorset, UK.

### Instrumentation

BioDrop *μ*LITE UV/visible spectrometer was purchased from Biochrom Ltd Cambridge, UK. Horiba JY FluorMax – 4 spectrofluorometer was purchased from Horiba UK Limited, Northampton, UK. The 6 kOe vibrating sample magnetometer (VSM) was built in-house at the University of Central Lancashire, Preston, UK. Nicolet AVATAR 330 FTIR spectrophotometer with Pike MIRacle accessory and FEI Tecnai 12 TEM at 100 kV with a Tietz F214 2k × 2k CCD camera were purchased from Thermo Fisher Scientific, Loughborough, UK. Bruker D2 Phaser Bench top X-ray diffractometer was purchased from Bruker UK Limited, Coventry, UK. Anton Paar monowave 200 microwave oven was purchased from Anton Paar Ltd Hertfordshire, UK. SLS Lab basics centrifuge was purchased from Scientific Laboratory Supplies, Nottingham, UK.

### Methods

#### Synthesis of fluorophore tagged MIPs

##### Solution preparation

A solution of 10% (w/v):10% (v/v) SDS:AcOH was prepared for use in the washing (protein elution) stages before the template reloading stage. SDS (10 g) and AcOH (10 ml) was dissolved in 990 ml of deionised (DI) water, to produce 1 l of the washing solution.

##### MIP preparation

Bulk MIP hydrogels were produced, using an optimized methodology [58], where a 10% cross-linking monomer/N,N′-methylenebisacrylamide hydrogel was found to produce the optimal imprint for Mb, in terms of specificity and rebinding efficiency of the MIP, compared with the non-imprinted polymer (NIP) [58].

##### Fluorescein MIP synthesis

Into an Eppendorf tube, 12 mg of myoglobin template was dissolved in 435 *μ*l of deionised water vortexed for 1 min, followed by the addition of 135 *μ*l (7.6 × 10^–4^ mol) of 40% AAm solution, 300 *μ*l (3.9 × 10^–5^ mol) mBAm (cross-linker), and 100 *μ*l of 1 × 10^–3^ mMol fluorescein *O*-acrylate solution (monomer: crosslinker: f-monomer molar ratio was 760:39:1), then vortexed for a further minute). Finally, 10 *μ*l of a 5% TEMED (v/v) solution and 20 *μ*l 5% APS (w/v) solution were added and the mixture was vortexed for 1 min. Solutions were purged with nitrogen for 5 min and polymerization occurred overnight at room temperature (∼20 °C). Corresponding NIPs were produced using the same method, but in the absence of a protein template.

After polymerization, the gels were granulated separately, using a 35 *μ*m sieve. The refined gels were washed with five 1 ml volumes of deionised water followed by five 1 ml volumes of 10% (w/v):10% (v/v) SDS:AcOH eluent; this allowed for the removal of the template protein from the MIP cavities. Following this, the gels were washed with five 1 ml volumes of deionised water to remove all residual 10% (w/v):10% (v/v) SDS:AcOH from the MIP gels. Each wash step was followed by centrifugation, whereby the gels were vortexed then centrifuged (using SLS Lab Basics centrifuge) for 5 min at 15 000 rpm (RCF: 15 100 × g). Corresponding NIPs were synthesized using the same procedure as the MIPs, but in the absence of the template molecule.

##### MIP rebinding studies

The subsequent protein rebinding ability of the conditioned and equilibrated MIPs and NIPs were characterized using the BioDrop *μ*LITE UV/visible spectrometer. Hydrogels (200 mg) were then each treated with 400 *μ*l of a 3 mg ml^−1^ myoglobin (template protein) solution. The polymer/protein solutions were mixed on a rotary vortex mixer and allowed to associate at room temperature (∼20 °C), then washed four times with 1 ml of deionized water. Each reload and wash step for hydrogels was followed by centrifugation for 5 min at 15 000 rpm (RCF: 15 100 × g). All supernatants were collected for analysis by spectrophotometry (at 405 nm for myoglobin).

Selectivity studies were performed on the MIP using the protein, bovine serum album (BSA). This performed using the previous method, but substituting the 400 *μ*l of a 3 mg ml^−1^ myoglobin (template protein) solution, with a 400 *μ*l of a 3 mg ml^−1^ bovine serum albumin (non-template protein) solution.

#### Myoglobin functionalized SPIONs

##### Preparation of functionalized SPION

The functionalized SPIONs were prepared through a one-pot solvothermal microwave method. FeCl_3_ˑ6H_2_O was used as a single iron source and glutaraldehyde as a modification ligand. With stirring, 0.5 g of FeCl_3_ˑ6H_2_O and 1.8 g of NaOAc were dissolved in 15 ml of ethylene glycol in a 30 ml Anton Parr G30 microwave reaction vial (MRV). The functionalization agent (glutaraldehyde (3.5 ml) was then added to the resulting solution and stirring continued for a further 5 min. The magnetic stirrer bar was then removed and the MRV was placed into an Anton Paar monowave 200 microwave oven and the reaction was heated up to a temperature of 200 °C with a ramp time of 2 min. The reaction was held at 200 °C for 20 min under pressure (9 bar). The resulting composite products were washed five times with deionised water followed by two washes of ethanol, and then collected with a magnet and finally dried for further use. The method was repeated, but in the absence of a functionalization agent, to produce bare SPIONs.

##### Protein conjugation to functionalized SPION

The protein adsorption capacity of the obtained magnetic particles was investigated using Mb as the model protein. Five Mb solutions of 0.2, 0.3, 0.4, 0.5, 0.6 mg ml^−1^ were prepared and 1 ml of each protein solution was added to 20 mg of the magnetic material. The mixture was vortexed followed by shaking. We determined that the optimum time for this step was 30 min. Afterwards, the mixture was then centrifuged (SLS Lab basics centrifuge) for 5 min at 15 000 rpm (RCF: 15 100 × g). The amount of protein adsorbed onto the SPIONs (functionalized and bare) was calculated through comparing the initial and final concentrations of protein remaining in the supernatant. The concentration of the non-adsorbed protein was measured by spectrophotometry (405 nm for Mb) using a BioDrop *μ*LITE UV/visible spectrometer.

##### SPION characterization

Functionalized nanoparticles were suspended in ultra-pure water (0.1 g in 50 *μ*l water) and a 5 *μ*l droplet was deposited onto a Formvar/carbon coated 200 mesh copper TEM grid (Agar Scientific, UK). After 1 min the grid was blotted, washed for 30 s in ultra-pure water, blotted again and allowed to dry. Images were collected using a FEI Tecnai 12 TEM at 100 kV with a Tietz F214 2k × 2k CCD camera. Fourier transform infrared (FTIR) spectra were obtained on a Nicolet AVATAR 330 FTIR spectrophotometer fitted with a Pike MIRacle accessory. The FTIR was recorded using transmission mode and the spectrum was collected at room temperature in 4000–400 cm^−1^ region with a resolution of 4 cm^−1^ using 32 scans. The crystal structure information was recorded using a Bruker D2 Phaser Bench top x-ray diffractometer system equipped with a LynxEye detector and using Cu K*α*_12_ radiation (*λ* = 1.5418 Å). Data were collected between 5° and 80° 2-theta using a step size of 0.02019°. Pawley fits were performed using the TOPAS program. Initial lattice parameters for ETS-10 and quartz were taken from Anderson *et al* [59] and le Page and Donnay [60], respectively. A 12-term Chebyshev background function and a pseudo-Voight peak profile function were all used for refinements. Magnetization curves were measured at room temperature using an in-house 6 kOe VSM. The samples were first crushed with a pestle and mortar, to break-up the large agglomerations of the dry state. This enabled them to be packed into rectangular glass microslides (Camlab) of internal dimensions 0.40 ± 0.04 mm by 4.0 ± 0.1 mm that were cut in lengths of 10.5 ± 0.5 mm. In this geometry the demagnetization factor (*N*), was kept low and within the range 0.030 < *N* < 0.068 [61].

#### F-MIP assay

A MIP-based non-competitive assay was designed for the detection of the AMI biomarker myoglobin. The assay uses an excess amount of F-MIP as a capture molecule, to bind to all of the myoglobin within the sample. Mb-SPIONs were then used to bind to all the remaining unbound MIP, which was then subsequently removed with magnetism. The MIP left in the sample was then removed and analyzed using fluorescent spectroscopy, with fluorescent intensity directly related to the amount of myoglobin in the sample.

##### Non-competitive assay calibration

Solutions containing known concentrations of Mb (6, 0.6, 6 × 10^–2^, 6 × 10^–3^, 6 × 10^–4^, 6 × 10^–5^, 6 × 10^–6^, 6 × 10^–7^, 6 × 10^–8^, and 6 × 10^–9^ mg) were prepared in 1 ml of PBS, followed by the addition of 100 mg of F-MIP/NIP to the sample. This was then vortexed for 30 s and left for 5 min to allow the protein to associate with the imprinted or non-imprinted gel. Next, 30 mg (optimum amount) of Mb-SPION was added, followed by further vortexing (30 s) and protein association (5 min). MIP/NIP particles bound to the Mb-SPION were removed using a magnet, and the supernatant then analyzed using a Horiba JY FluorMax – 4 spectrofluorometer with an excitation at 490 nm and an emission scan range of 200–900 nm. A calibration curve was created by plotting the log of the amount of protein loaded (mg ml^−1^) versus log intensity (CPS). The calibration was repeated, but with FCS used instead of PBS.

To test the assay, 1 ml samples of FCS were spiked with known concentrations of Mb (10, 80 and 160 ng ml^−1^). These values were chosen because it would allow for the detection of Mb within the normal range of healthy patients, as well as showing the range on and above the borderline for elevated levels (above 85 ng ml^−1^) seen in patients [11]. Next, 100 mg of F-MIP/NIP was added to the sample and vortexed for 30 s and left for 5 min to allow the protein to associate with the imprinted gel. Next, 30 mg (optimum amount) of Mb-SPION was added, followed by further vortexing (30 s) and protein association (5 min). MIP particles bound to the Mb-SPION were removed using a magnet, and the supernatant then analyzed using fluorescence spectroscopy with excitation at 490 nm and an emission scan range of 200–900 nm. The results were compared with the calibration plot in order to evaluate the error within the assay.

## Results and discussion

### MIP synthesis and rebinding studies

Fluorophoric MIPs (F-MIPs) and their corresponding NIPs were successfully synthesized using the optimized method [58]. Rebinding studies were performed on these and the results are presented in table 1. This confirmed that there was no detrimental effect in the ability of target molecule to bind to MIP when integrating the fluorophore into the MIP/NIP synthesis.

**Table 1.** Percentage of the myoglobin target protein (Mb) rebind to the acrylamide-based MIP and NIP with incorporated fluorophore and the corresponding imprinting factor as well as percentage of bovine serum albumin non-target protein (BSA) bind to MIP.

Fluorophore	MIP percentage of Mb rebind (%)	NIP percentage of protein bind (%)	Imprinting factor (IF)	MIP percentage of BSA bind (%)
Fluorescein *O*-acrylate	85.8 ± 2.9	43.9 ± 2.7	1.95	42.4 ± 2.3

Our previous work, showed that a polyacrylamide-based MIP for the target protein myoglobin had a rebinding capacity , of 85.4% with an IF value of 1.80, suggesting that the MIP has a good degree of affinity with a high degree of selectivity compared to the corresponding NIP and other acrylamide-based functional monomers [57]. When the fluorophore was integrated into a polyacrylamide-based MIP, the amount of myoglobin target that rebound was 85.8%. This shows that the fluorophore integrated MIPs produce rebinding characteristics which are very similar to that of the polyacrylamide MIP (±1.6%) [57], indicating that there is little to no effect on rebinding of the target protein, when integrating the fluorophore into the MIP. These results offered assurance that a fluorophore integrated MIP can be used to develop a fluorometric MIP-based assay for myoglobin. Table 1 also shows that the Mb templated MIP will bind 42.4% of non-specific BSA. This value is very similar to the binding capacity of the NIP, confirming that the Mb MIP has selectivity towards the target protein.

### Rapid microwave-assisted synthesis of functionalized SPIONs

Microwave-assisted synthesis is based on the accelerated heating of materials due to dielectric heating effects. The microwave energy produced is only transferred directly to those reaction components, which are susceptible to microwave polarization. This improves the energy efficiency, by only heating the reaction mixture and reduces the need to heat any reaction vessels, unlike conventional heating. As a result of this directed heating, heating the reagents is much faster than with conventional methods. This, in turn, minimizes the time it takes for the reaction to reach its activation energy and can radically reduce the reaction time, with the added benefit that this method reduces unwanted side reactions and by-products [62].

Ethylene glycol was the solvent used in this reaction and is known to have a high dissipation factor (tan *δ* = 1). The solvent has a higher capacity to absorb microwaves and convert to thermal energy, making it ideal for microwave synthesis [63]. Producing functionalized SPIONs by a conventional solvothermal method [48] takes 480 min for the reaction to be complete, whereas our microwave-assisted method only takes 20 min. Different synthesis times were trialed, before settling upon 20 min; 10 min did not yield nanoparticles. Whereas 30 min was successful in producing nanoparticles, we selected 20 min synthesis for further studies.

**Figure 1.**
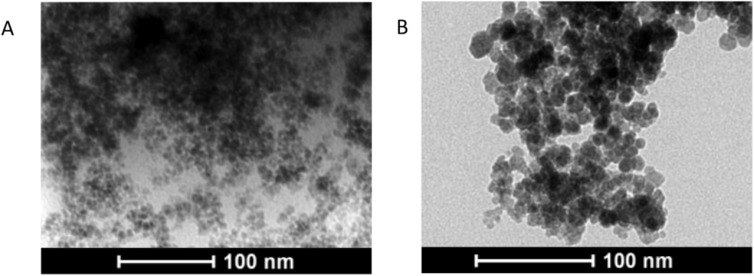
TEM images of SPION@CHO (A) and SPION (bare) (B). Images were collected using a FEI Tecnai 12 TEM at 100 kV with a Tietz F214 2k × 2k CCD camera.

The TEM images for SPION@CHO, and SPION (bare) are shown in figures 1(A), and (B), respectively. The obtained nanoparticles are nearly spherical and dispersive with a diameter, ranging from 1.6 to 11.6 nm. The microwave method, generally produces particles that are smaller (7.5 and 7.4 nm, for SPION@CHO and SPION (bare) respectively) than the particles produced using a solvothermal method (19.3 nm) [48], with a summary of all the particles shown in table 2. The SPION@CHO (figure 1(A)) appears to show small agglomerates of SPIONs embedded within a polymeric matrix, which could account for the larger size of the individual particles within each agglomerate. The bare SPIONs (figure 1(B)) shows particles that appear consistently spherical shape and similar in size.

**Table 2.** A comparison for the reaction methods to produce *functionalized SPION* between a conventional solvothermal method versus our microwave method.

	Reaction time (min)	Average particle size (nm)	Standard deviation (nm)
Solvothermal method [48]	480	19.3	1.8
Microwave method (CHO)	20	7.5	1.8
Microwave method (bare)	20	7.4	1.4

**Figure 2.**
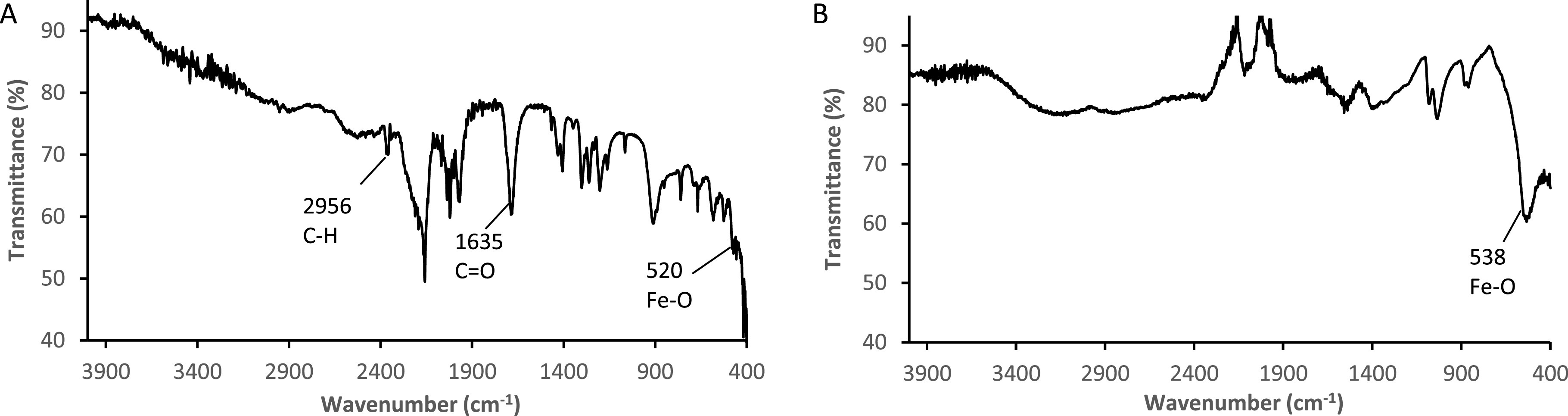
FTIR spectra of SPION@CHO (A), and SPION (bare) (B), obtained using a Nicolet AVATAR 330 FTIR spectrophotometer fitted with a Pike MIRacle accessory.

**Figure 3.**
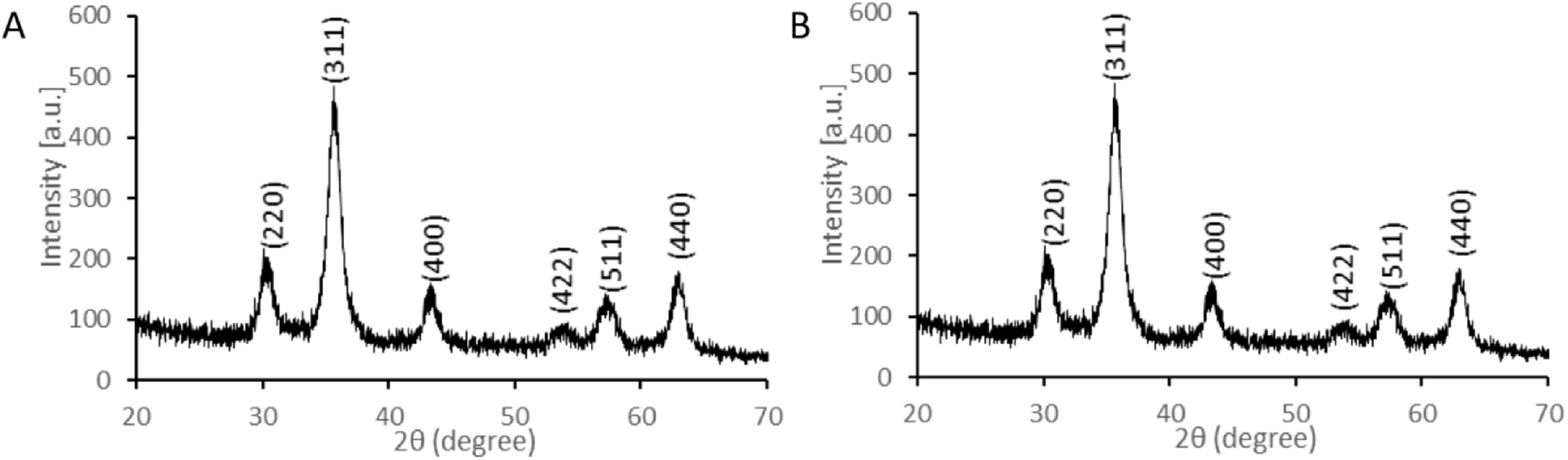
XRD pattern of SPION@CHO (A) and SPION (bare) (B), obtained using a Bruker D2 Phaser Bench top x-ray diffractometer system equipped with a LynxEye detector and using Cu K*α*_12_ radiation (*λ* = 1.5418 Å).

The FTIR spectra of the functionalized SPIONs are shown in figures 2(A) and (B), where the main functional groups of the predicted structure can be observed. Figure 2(A) shows the FTIR spectrum for the aldehyde functionalized SPION (SPION@CHO). The absorption band at 520 cm^−1^ is assigned to the Fe–O stretching vibration. The peak at 1635 cm^−1^ is assigned to the stretching vibration of carbonyl, while the typical peak at 2956 cm^−1^ is due to the asymmetric stretching of C–H vibration. These characteristic peaks indicate that iron oxide magnetic particles containing aldehyde groups on the surface have been obtained through our one-pot microwave facilitated solvothermal method [48]. Figure 3(B) shows an absence of any functional groups peaks, with the only peak observed being the absorption band at 538 cm^−1^, which is assigned to the Fe–O stretching vibration. The absence of any other peaks again highlights that fuctionalization has taken place in figure 2(A).

The XRD patterns for the synthesized SPIONs and functionalized SPIONs produce the same six strong diffraction peaks, which is to be expected as XRD only determines the atomic and molecular structure of a crystal and does not include any information on the functionalization. These XRD patterns are illustrated in figure 3, where the six strong diffraction peaks for the sample are observed in the 2*θ* range of 20°–80°, which are indexed as (220), (311), (400), (422), (511), and (440), respectively. Figures 3(A) and (B) can be seen to be very similar in nature and this is shown in the analysis of the XRD data. The crystallite size of the of the SPIONs was estimated using the Scherrer equation (equation (1)), which relates the crystallite size (*D_p_*) and a specific diffraction peak broadening [64, 65]:

\begin{eqnarray*}{{D}}_{p}=\displaystyle \frac{{K}\lambda }{{\beta }_{311}}\,\cos \,{\theta }_{311},\end{eqnarray*}

where, *D*_*p*_ is the average crystallite size, *K* is the Scherrer constant (0.94), *β*_311_ is line broadening (full-width at half maximum, in radians) and *θ*_311_ is the Bragg angle (radian). The estimated crystallite sizes for the Fe_3_O_4_ NPs are 7.47 nm and 7.45 nm for the SPION@CHO and SPION (bare), respectively, and is consistent with the size estimates using TEM (table 2). The lattice parameters ‘*a*’ were determined to be 8.3656 and 8.3547 for the SPION@CHO and SPION (bare), respectively, with these values being similar to those found in literature (*a* = 8.399) [66]. These results are consistent with iron oxide found in the inorganic crystal structure database (ICSD Collection Code 5247) [67], and confirms that we have produced Fe_3_O_4_ (magnetite) [68, 69].

**Figure 4.**
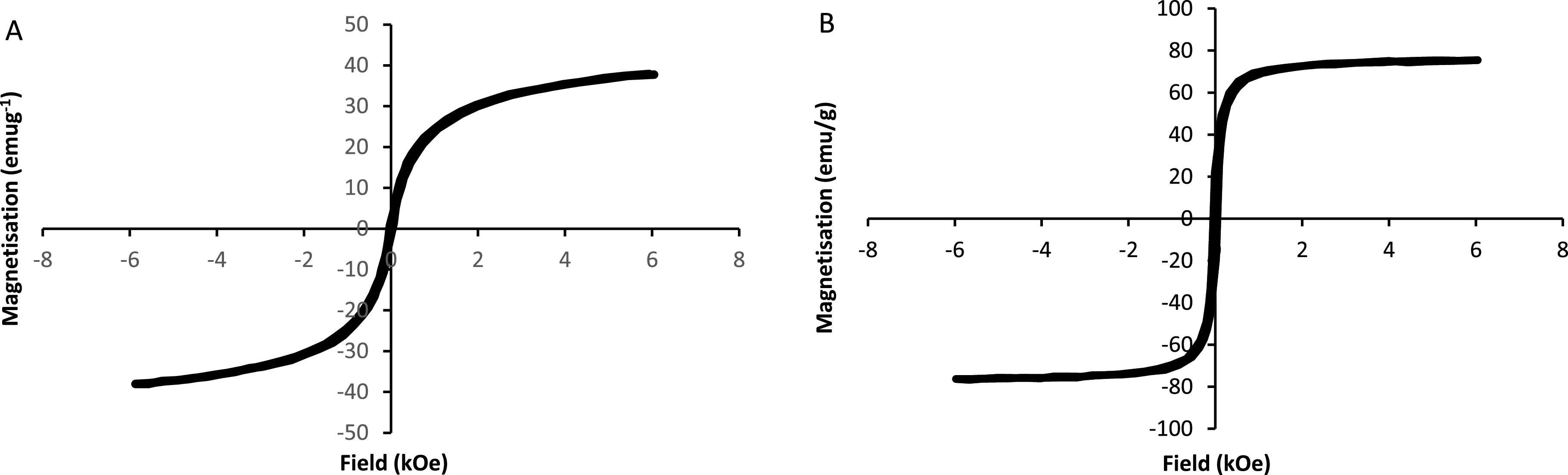
VSM spectra of SPION@CHO (A) and SPION (bare) (B), measured at room temperature using an in-house 6 kOe Vibrating Sample Magnetometer.

The magnetic properties of the functionalized SPIONs were studied using a VSM at room temperature (figures 4(A) and (B)). The magnetization curves with S-like shape are symmetrical to the origin and there is near-zero hysteresis, with both remanence and coercivity approximately zero, indicating that the samples are predominantly superparamagnetic. The saturation magnetization values of 38 emu g^−1^ (SPION@CHO) and 75 emu g^−1^ (SPION (bare)) are lower than the ferrimagnetic bulk value of 92 emu g^−1^ [70] and is expected when in a superparamagnetic state at particle sizes <100 nm, as is the case here [71]. However, the values are still high enough for easy and rapid separation when under an external magnetic field. The TEM image of the functionalized SPIONs (figure 2(A)) shows the magnetic particles are distributed within the nonmagnetic functionalized material and hence this dilutes the magnetic content fraction within the sample volume when compared to the bare SPIONs and will contribute to the lower saturation magnetization value observed [72].

**Figure 5.**
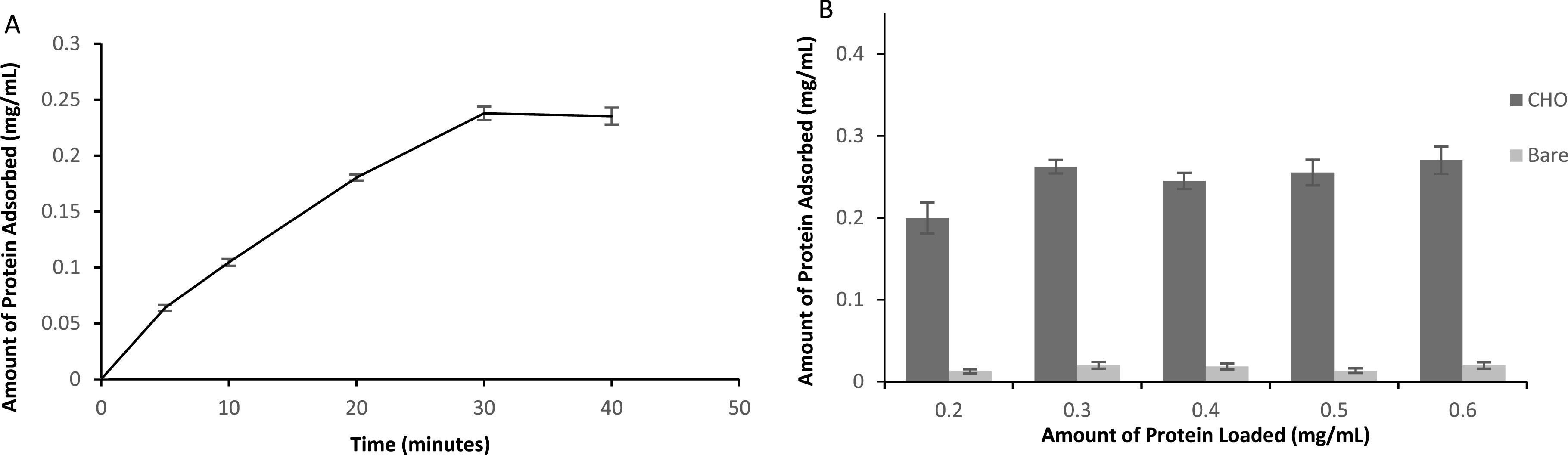
Optimum adsorption time for Mb binding to 20 mg of SPION@CHO particles (A) and adsorption capacity (B) of the SPIONs. Measured using spectrophotometry (405 nm for Mb) using a BioDrop *μ*LITE UV/visible spectrometer.

We further investigated the ability of these particles to covalently bind protein. This was with a view to use protein tethered SPIONs in the development of our F-MIP based protein diagnostic. Protein adsorption onto the SPION@CHO was investigated by adding 1 ml of known concentrations of a myoglobin solution. Prior to this, we had determined that a minimum of 30 min was required for optimum adsorption of 0.4 mg ml^−1^ of protein (figure 5(A)). The results showed that on average, 20 mg of SPION@CHO was able to adsorb 0.247 ± 0.013 mg of myoglobin, respectively (figure 5(B)). In contrast, binding of protein on non-functionalized SPIONs, showed only limited protein adsorption (0.012 ± 0.004 mg). This suggests that the mechanism for protein attachment to the aldehyde functionalized SPION is primarily through covalent binding; glutaraldehyde has been used extensively in the immobilization of proteins [73]. The high reactivity of glutaraldehyde towards proteins is based on the presence of several reactive residues in proteins and molecular forms of glutaraldehyde in aqueous solution, which may lead to many different possible reaction mechanisms, including, but not limited to, aldol condensation or Michael-type addition [74].

### Optimization of protein-SPION level for assay development

The fluorescently tagged MIP and NIP were explored using fluorescent spectroscopy to determine if protein binding had an effect on the fluorescent emission of the sample. As can been seen in figure S1, in the supplementary data (available online at stacks.iop.org/NANO/32/095502/mmedia), the results show that the F-MIP has an emission intensity value which is lower than that of the F-NIP. This is to be expected as a F-MIP molecule contains cavities, resulting in less polymer matrix within the same volume of particle (35 *μ*m particle), meaning there is less polymer within the F-MIP for the light to be absorbed, therefore resulting in less photons being emitted; consequently, lowering the emission intensity. Polymer matrix differences between MIP and NIP were explored in the work of Larpant *et al* (2019) who showed that the MIP contained a matrix that was highly absorbent, with strong affinity of the target molecule [75]. The resulting changes in polymer matrix explains the changing in absorbance, with work by Pelras *et al* (2017) showing changes to a hydrogel matrix can increase transparency [76]. The fluorescence spectra (figure S2), for all concentrations of the fluorescein *O*-acrylate monomer, show a peak at 482 nm, which is due to excitation of the sample. The energy from the emitted photon is responsible for the peak seen at 514 nm. As the concentration of the fluorescein monomer is diluted further the intensity of the peak at 514 nm steadily decreases as to be expected, with the fluorescence intensity being proportional to the concentration of the fluorophore.

In constructing the non-competitive MIP-based assay for the detection of the AMI biomarker Mb, aldehyde functionalized SPIONs (SPION@CHO) were used, with myoglobin (Mb) bound to the nanoparticle (Mb-SPION). These nanoparticles are nearly spherical and dispersive with a diameter, averaging 7.5 nm, with a saturation magnetization value of 38 emu g^−1^, meaning they can be easily and rapidly separated from suspension under an external magnetic field.

**Figure 6.**
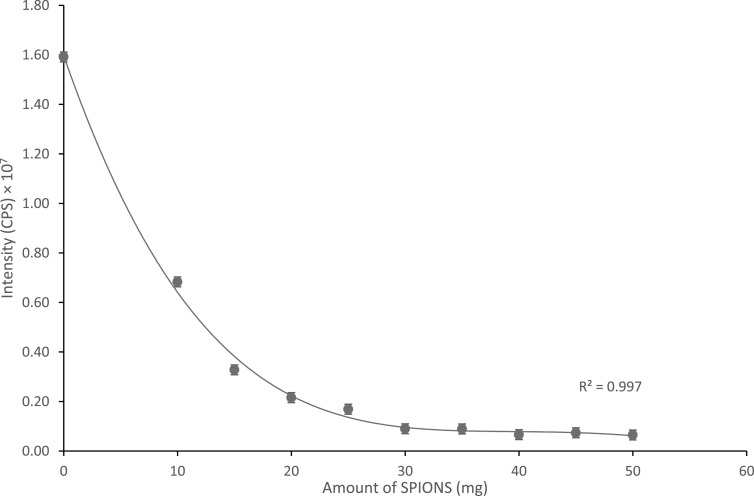
A plot showing how much Mb-SPION is needed to bind and remove all of the fluorescently tagged MIP from a 1ml 1:10 solution of fluorescein O-acrylate (1 × 10^–3^ mMol) MIP:H_2_O.

The amount (30 mg) of the Mb-SPION conjugate needed to bind to all of the F-MIP was first determined. This would then give a value for the maximum amount of Mb-SPION required per assay, and would simulate the situation of there being zero target Mb in the test solution. Masses (0–50 mg) of Mb-SPION were loaded into 1 ml of water containing 100 mg of fluorescein *O*-acrylate (1 × 10^–3^ mmol) MIP. A magnet was used to remove the F-MIP bound to the Mb-SPION complex. Any remaining unbound MIP left in solution was analyzed by fluorescence spectroscopy. The limiting amount of Mb-SPION needed to bind to all of the F-MIP was determined as the point when the measured fluorescence emission intensity petered out and remained constant (at about the same intensity as when measuring water only). Figure 6 shows that as the amount of Mb-SPION is increased, the fluorescence emission intensity of the sample decreases. This pattern is of exponential decay resulting from an initial rapid decrease in intensity, which steadily reaches a minimum at and above 30 mg of Mb-SION with an intensity of 734 490 CPS, which is of similar intensity of a sample of water only. It was determined that 30 mg of Mb-SPION was sufficient to bind all of the F-MIP in the solution, so this amount was selected be used within the assay.

### Non-competitive assay calibration

**Figure 7.**
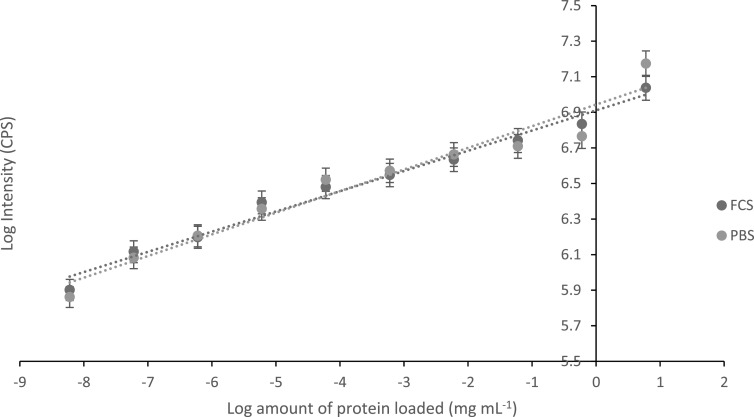
Calibration plots for the non-competitive assay, by comparing the log amount of protein loaded (mg ml^−1^) in fetal calf serum (FCS) (blue) and phosphate buffered saline (PBS) (orange), with the log fluorescence emission intensity (CPS).

Using a non-competitive assay format, a calibration was undertaken by dissolving known concentrations of target protein Mb (6, 0.6, 6 × 10^–2^, 6 × 10^–3^, 6 × 10^–4^, 6 × 10^–5^, 6 × 10^–6^, 6 × 10^–7^, 6 × 10^–8^, and 6 × 10^–9^ mg) into FCS (1 ml). FCS was used, with known Mb concentrations dissolved, in order to determine the selectivity and biocompatibility of the F-MIP, investigating whether non-target molecules present within the serum would interfere with the assay, leading to the production of false positives. FCS contains many different proteins (BSA, Immunoglobulin G (IgG), fibrinogen, transferrin, amongst others), with the main constituent being albumin, [77]. The F-MIP was added to the sample, with the target protein being allowed to associate with the MIP for 10 min. Next, 30 mg of Mb-SPION was added to the sample and further associated with any unbound MIP was allowed for 10 min MIP particles bound to the Mb-SPIONs were removed using a magnet, and the supernatant then analyzed using fluorescence spectroscopy. Calibration plots were created by plotting the log_10_ of the amount of protein loaded (mg ml^-1^) versus log_10_ intensity (CPS) and are shown in figure 7. The calibration plots show that the assay has a linear range with a lower LOD of 60 pg ml^−1^. This is a vastly wider range than that needed for the detection of Mb in patient samples (0–80 ng ml^−1^). That we can use this MIP-based assay to measure protein levels down to pg ml^−1^, makes it highly suitable for the potential development of a suite of assays for a range of biomarkers which require a low limit of detection including cancer markers such as PSA (<4 ng ml^−1^), CEA (<2.5 ng ml^−1^), and AFP (<10 ng) [11, 78]. Calibrations were produced in PBS buffer and FCS showing that the assay is specific only for the target protein Mb and shows minimal interference from other proteins (native to serum).

### F-MIP-based assay for analysis of samples

The MIP-based assay was further tested by spiking 3 × 1 ml samples of FCS with known concentrations of Mb (10, 80 and 160 ng ml^−1^). The results of the spike serum test are shown in table 3.

**Table 3.** Analysis of Mb spiked serum concentration using the developed non-competitive assay.

Mb spiked concentration (ng ml^−1^)	Fluorescent emission intensity (CPS)	Amount of Mb calculated in sample (ng)	Percentage recovery (%)
10	2164570 ± 1180	9.8	97.79
80	2782603 ± 1125	85.0	93.66
160	2970330 ± 1210	149.0	93.31

The results in table 3 show that when FCS is spiked with known concentrations of the AMI biomarker Mb, the MIP-based non-competitive assay was able to detect the Mb to within an error of 6.7%. This is an acceptable percentage error as it is similar to the percentage error of other assays which are commercially available and currently being used [14]. A comparison of these assays with the one developed is shown in table 4.

**Table 4.** A comparison of different assays available against the developed MIP-based assay.

Assay	Percentage error (%)
Developed MIP Assay	2.2–6.7
Access^a^	6.0–11.0
Hitachi^a^	3.8–5.8
Stratus^a^	3.4–6.5

^a^ Data for these assay kits supplied by the work of Zaninotto *et al* (2000) [14].

Table 4 shows that the developed MIP assay performs slightly better (with a percentage error range of 2.2%–6.7%) than the Access assay (with a percentage error range of 6.0–11.0). The results also show that the developed MIP assay performs very similarly to the Hitachi (with a percentage error range of 3.8%–5.8%) and Stratus (with a percentage error range of 3.4–6.5). This shows that the developed MIP assay is competitive with those that are currently on the market. All the market assays (Access, Hitachi, and Stratus) use Mb antibodies as the capture molecule [79].

**Figure 8.**
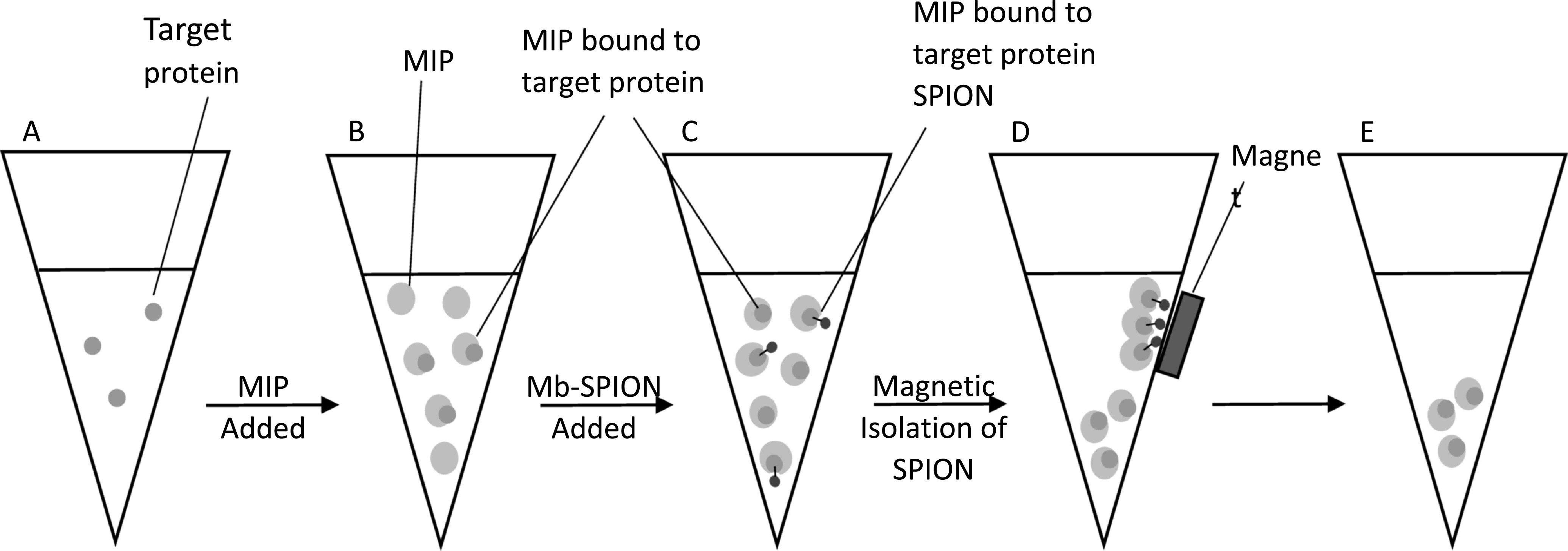
A schematic showing how the non-competitive assay works: (A) is the sample of target protein. (B) Excess amount of the fluorescently tagged MIP is added and binds to all the target protein. (C) Excess amount of target protein-SPION is added, this binds to all of the unbound MIP in the sample. (D) MIP bound to target protein-SPION is removed by magnetism. (E) After removal of MIP bound to the target protein-SPION, MIP bound to the target protein in the sample is left to be analyzed.

This uniquely developed MIP assay uses a fluorescence spectroscopy for detection of myoglobin from a spiked sample. Fluorescently tagged MIP is added to the sample containing the target molecule, whereby the target molecule would bind to the MIP. Excess MIP would then be removed by the addition of the myoglobin modified SPIONs, produced using the unreported microwave method that synthesized aldehyde functionalized SPIONs in 20 min. Previous studies synthesized these particles using non-microwave solvothermal synthesis, by either a one-pot synthesis or multi-step synthesis, often taking in excess of 8 h to produce [48, 52, 53]. This novel synthesis dramatically decreases the time needed to produce functionalized SPIONs (SPION@CHO), thus using less energy and reagents, and making this synthesis much greener than traditional methods. These particles would bind to the excess MIP, which could then be separated using a magnet leaving only the target protein bound MIPs in suspension. The F-MIPs in suspension could then be analyzed using fluorescence spectroscopy, where by the intensity of the signal is proportional to the amount of target protein in the sample, as shown in figure 8.

## Conclusion

In this work, we have demonstrated a microwave facilitated, one-pot solvothermal method for the synthesis of functionalized SPIONs. This method allowed for the rapid synthesis of functionalized SPIONs with time to production being reduced from many hours to a mere 20 min This significant reduction in synthesis time, shows that functionalized SPION particles can be produced using much less energy than previous solvothermal synthesis methods. A non-competitive MIP-based fluorometric assay was subsequently developed for the AMI biomarker Mb. The assay used a fluorophore tagged MIP as a capture molecule, binding specifically to myoglobin, enabling its detection and quantitative analysis. Calibration of the assay was conducted in FCS showing that the assay was specific only to the target protein. The assay has a low LOD, capable of detecting down to 60 pg ml^−1^ of Mb, suggesting the assay would be suitable for detection of Mb within patients where 0–80 ng ml^−1^ is the normal range for Mb levels in healthy patients and elevated levels (>80 ng ml^−1^) are seen in patients where AMI has occurred Analyzing FCS spiked with known concentrations of Mb demonstrated that our MIP-based assay can detect Mb within a biological sample with an error range of only 2.2%–6.7%. This is a similar range to antibody-based assay kits that are available on the market [14] and demonstrating that our developed MIP-based assay is able to compete with assay kits that are already available, but at a fraction of the cost. MIP-based protein assays can be a real alternative to antibody-based assays with major implications in the future development of disease diagnostics, especially in instances where antibodies are either difficult to produce or do not currently exist.

## Conflicts of interest

There are no conflicts to declare.
